# Preliminary validation of developmental weight suppression in youth with transdiagnostic eating disorders

**DOI:** 10.1186/s40337-025-01349-0

**Published:** 2025-07-31

**Authors:** Simar Singh, Erin E. Reilly, Catherine R. Drury, Alan Duffy, Philip S. Mehler, Erin C. Accurso, Kianna Zucker, Naomi Lynch, Daniel Le Grange, Renee D. Rienecke, Sasha Gorrell

**Affiliations:** 1 Department of Psychiatry and Behavioral Sciences, Univeristy of California San Francisco, San Francisco, CA USA; 2 Eating Recovery Center and Pathlight Mood & Anxiety Center, Denver, CO USA; 3 Acute Center for Eating Disorders at Denver Health, Denver, CO USA; 4 University of Colorado School of Medicine, Denver, CO USA; 5 Philip R. Lee Institute for Health Policy Studies, San Francisco, CA USA; 6 Department of Psychiatry and Behavioral Sciences, The University of Chicago, Chicago, IL USA; 7 Department of Psychiatry and Behavioral Sciences, Northwestern University, Chicago, IL USA

**Keywords:** Eating disorders, Weight suppression, Adolescence

## Abstract

**Background:**

Weight suppression (WS), traditionally defined as the difference between highest past and current weights at adult height, is a correlate and predictor of eating disorder (ED) psychopathology. However, for growing adolescents, it may be more appropriate to use a developmentally-adjusted calculation of WS. This study compared how developmental WS, calculated using *z*BMIs, compared with traditional WS, calculated using weights, as correlate of ED psychopathology in treatment-seeking adolescents with transdiagnostic EDs.

**Methods:**

Adolescents with EDs (*N =* 93) completed the Eating Disorder Examination Questionnaire (EDE-Q) at presentation to outpatient care. Weight histories were extracted from medical records. Regressions examined the association between each measure of WS and EDE-Q scores, adjusting for ED diagnosis. Dominance analyses with bootstrapping assessed whether developmental WS outperformed traditional WS.

**Results:**

Developmental WS negatively associated with EDE-Q Shape (*sr*^*2*^ *=* 0.05, *p =*.020) and Weight Concern (*sr*^*2*^ *=* 0.05, *p =*.021). In contrast, traditional WS did not associate with any EDE-Q scores. Although dominance weights were larger for developmental WS compared to traditional WS, bootstrap sampling revealed no significant differences in magnitudes.

**Conclusions:**

Results preliminarily support developmental WS as a correlate of body image concerns in youth with EDs, though replication is needed.

## Abstract (plain language summary)

Weight suppression (WS), traditionally calculated as the difference between highest past and current weights, predicts transdiagnostic eating disorder (ED) onset and positively correlates with ED symptoms. However, a simple subtraction of weights does not capture differences with respect to height, age, and sex that may impact WS’s associations with ED pathology. This is a particular concern for youth, who are growing in age and height. For instance, 8 kg of WS may have a different impact on a 10-year-old who is 142 cm versus an 18-year-old who is 172 cm. To address this shortcoming, a “developmentally-adjusted” calculation of WS that quantifies the difference between highest premorbid and current BMI *z*-scores was recently published. The present study is the first to quantitatively compare how traditional versus developmentally-adjusted calculations of WS differentially associate with baseline ED pathology in treatment-seeking youth with transdiagnostic EDs. In contrast to the traditional calculation of WS, which did not associate with any ED symptoms, we found that the developmentally-adjusted calculation negatively associated with global eating pathology and body image concerns. Our findings provide quantitative evidence that a z-BMI-based calculation of WS, as opposed to a weight-based calculation, may be a better correlate of ED symptomatology in youth.

## Background

Weight suppression (WS), defined as the difference between one’s current and highest past weights since reaching adult height [1], reliably associates with eating disorder (ED) psychopathology. Across the lifespan in individuals with EDs, higher WS positively associates with fear of weight gain, dieting, duration of illness, and relapse risk, among other outcomes [2]. Longitudinal work has also found that WS predicts the onset of transdiagnostic EDs over three years [3], “bulimic syndrome” over ten years [4], and bulimia nervosa (BN) over 20 years [5] – even after accounting for other relevant predictors (e.g., dietary restraint, body image concerns). Although more research is needed to clarify the mechanisms by which WS exerts these effects, these data suggest that WS may play a role in the development and maintenance of EDs.

Despite these associations, several researchers [6–8] have highlighted potential methodological concerns regarding the calculation of WS as a difference in weights [1], arguing that a simple subtraction of raw weights does not capture differences relative to height, age, and sex that may moderate the impact of WS on ED symptoms [8]. Moreover, traditional WS presents a variety of developmental confounds given its assumption that adult height has been reached. This precludes its use in youth, who are growing in height and undergoing sex-specific pubertal changes. For example, WS of 4.5 kg may have distinct clinical and metabolic implications for a 7-year-old versus a 16-year-old, or for a premenarchal cisgender girl versus an age-matched cisgender boy. In another example, a patient who is 10 years old and fails to gain weight by age 12 would have WS of 0 kg. This value would suggest little to no clinical concern but fails to capture the lack of normative weight gain that could be secondary to disordered eating and/or contribute to ED risk.

To address these shortcomings, several authors adopted a *z*-BMI (i.e., BMI *z*-score) approach to calculate WS among youth [9–11], a method formally operationalized by Singh and colleagues [8] as “developmental WS.” By using *z*-scores, WS can be equally compared across individuals of different ages, heights, and sexes. Studies using a developmental calculation of WS found that higher WS related to worse symptomatology and predicted poorer treatment outcomes among adolescents with anorexia nervosa (AN), including lower weight at end-of-treatment and follow-up [11], higher levels of ED pathology at follow-up [10], and likelihood of ED persistence at follow-up [10]. Developmental WS is also associated with ED features in youth with BN, including more frequent binge eating, longer duration of illness, and greater weight and shape concerns [9]. 

Compared to traditional WS, developmental WS has demonstrated construct and incremental validity along psychological [12, 13] and biological outcomes [14, 15] among treatment-seeking *adults* with BN. Though adults may still benefit from developmental WS’s adjustment for age, sex, and height (e.g., consider the impact of 5 kg WS on a person who is 185 cm versus 150 cm tall), its incremental utility for adolescents important and critically missing from the current literature. Prior work indicates that a *z*-BMI approach to WS predicts ED outcomes in youth with AN and BN [9–11, 16], yet no work has compared how traditional and developmental calculations of WS differ in their associations with ED symptoms in adolescents. Clarifying whether WS in adolescence is best calculated using weights or *z*-BMIs can inform (1) research practices for calculating WS in this population and (2) clinical practices for selecting a measure of WS most indicative of clinical impairment.

To examine the incremental validity of developmental WS in youth, this methodological study aimed to contrast associations between traditional and developmental calculations of WS with symptoms in a sample of adolescents presenting for outpatient treatment with transdiagnostic EDs. Based on prior work [2], we hypothesized that developmental WS would: (1) positively associate with ED psychopathology and (2) be a stronger correlate of ED severity than traditional WS.

## Methods

### Participants

**Fig. 1:**
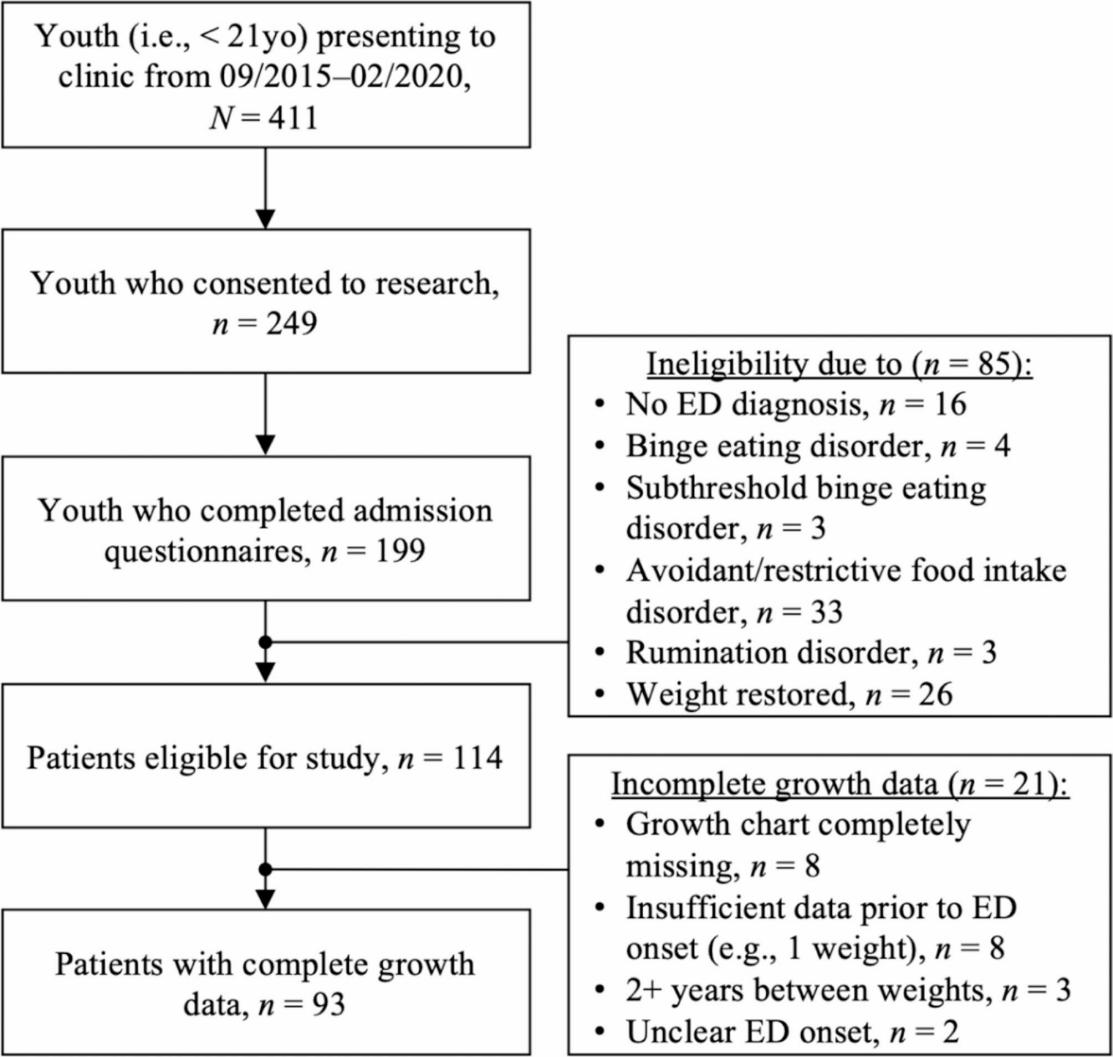
Flowchart of patients eligible for current study

Participants were youth (i.e., < 21 years old) presenting to outpatient ED treatment at an academic medical center in the United States (September 2015 to February 2020). To be included in this retrospective analytic study, individuals needed to have an ED diagnosis. Participants were excluded from the analytic sample if they: failed to complete admission questionnaires (*n* = 50); did not have an ED (*n* = 16); were diagnosed with an ED without body image concerns (e.g., avoidant/restrictive food intake disorder, rumination disorder; see Fig. 1), given the EDE-Q may not be valid for such presentations [17] (*n* = 43); were weight restored upon admission (*n* = 26); or had incomplete growth chart data (*n* = 21).

A total of 93 patients were included. This study was approved by the site’s Institutional Review Board. Parents or legal guardians provided informed consent for participation, and patients provided assent (or consent if ≥ 18 years).

### Procedures

Licensed mental health clinicians conducted assessments, informed by the *DSM-5* [18], to determine ED diagnoses. All patients completed demographic and self-report questionnaires regarding symptoms at admission. Patients self-reported date of ED onset, which was used to circumscribe the premorbid vs. postmorbid periods.

Growth chart data were either entered electronically or hand-plotted and scanned into patients’ electronic medical records. Patient medical records were then mined for the following: weight corresponding to their highest premorbid BMI percentile; height corresponding to their highest premorbid BMI percentile; and age corresponding to their highest premorbid BMI percentile. Current weight, height, and age were reported upon intake. Growth charts were considered incomplete if data were either missing entirely or partially – for example, missing data for significant periods (e.g., a gap of two or more years between data points) or an insufficient number of data points (e.g., ≤ 3 data points prior to ED onset).

### Measures

#### Eating disorder examination questionnaire (EDE-Q)

The EDE-Q [19] is a 28-item self-report questionnaire that assesses ED-related cognitions and behaviors over the past four weeks. It generates four subscales (Restraint, Eating Concern, Shape Concern, Weight Concern) that yield an averaged Global Score. Scores range from 0 to 6, with higher scores indicating more severe pathology. All subscales demonstrated excellent internal consistency in the current sample (Restraint, 𝛼 = 0.90; Eating Concern, 𝛼 = 0.80; Shape Concern, 𝛼 = 0.96; Weight Concern, 𝛼 = 0.90).

#### Traditional WS

Because study participants may have not yet reached adult height, traditional WS, as described here, represents a deviation from its usage in prior literature. Here, traditional WS was calculated by subtracting an individual’s current weight from their highest past weight, regardless of height. Highest past weights were extracted from patient growth charts. Following guidelines for calculating WS [1], all negative values were recoded to zero, as negative values indicate an individual is not weight suppressed.

#### Developmental WS

Developmental WS was calculated by subtracting an individual’s current *z*-BMI from their highest premorbid *z*-BMI using available growth chart data. Data collected from patient growth charts included: [1] individuals’ BMIs corresponding to their highest premorbid percentile, and [2] the ages at which these BMIs were reached. These values were entered into an online calculator (https://niuxin.shinyapps.io/devws/),(8) which calculates highest premorbid and current *z*-BMIs from respective BMIs, ages, and sex, subtracts current *z*-BMI from highest premorbid *z*-BMI, and recodes all negative values to zero.

### Data analytic strategy

#### Hypothesis testing

Analyses were conducted in RStudio, v.2023.09.1. Linear regressions tested the independent effects of each measure of WS on EDE-Q admission scores. Given our primary aim was to test incremental validity of developmental over traditional WS [20], models only controlled for diagnostic heterogeneity. Diagnosis was dummy-coded, with low-weight AN (AN restricting type, AN binge-purge type) as the reference diagnosis.

Dominance analyses (‘dominanceanalysis’ package [21]) tested whether developmental WS outperformed traditional WS in its strength of relation to ED symptoms [22–26]. Unlike relative weights analyses, another approach that compares the unique contribution of independent variables in predicting a given outcome, dominance analyses are suited for hypotheses that probe whether one predictor outperforms another in the amount of variance explained [27]. Additionally, unlike standard linear regressions, where correlation across predictors is an issue, dominance analyses are robust to multicollinearity [27]. 

Because the sampling distribution of dominance weights is unknown, bootstrap resampling (‘boot’ package [28]) determined whether the weights for developmental WS were significantly greater than those for traditional WS, consistent with recommendations in prior work [29]. We began by creating 1,000 bootstrap samples from the original dataset. For each sample, linear models that included both measures of WS were fitted, separate coefficients for developmental WS and traditional WS were extracted, and the difference between the coefficients for developmental WS and traditional WS was computed. We then calculated 95% confidence intervals for the distribution of the coefficient differences. If the confidence interval included 0, then the weights for developmental WS and traditional WS were not significantly different.

#### Power analysis

Power analyses were conducted in G*Power [30]. With significance criterion 𝛼 = 0.05, our sample size of *N* = 93 provided 97.2% power to detect a medium effect, similar to prior work (*d* = 0.48) [12], for associations between WS and EDE-Q scores.

Because no published research has tested developmental WS relative to traditional WS using dominance analyses, power analyses were conducted to estimate expected effect sizes at significance criterion 𝛼 = 0.05. Our sample size of *N* = 93 provided 80% power to detect a medium effect (*d* = 0.54) for dominance analyses testing the relative strength between traditional and developmental WS with respect to EDE-Q scores.

## Results

Participants were mostly White (*n* = 72, 77.4%), cisgender females (*n* = 79, 84.9%). Average traditional WS was 5.48 kg (*SD* = 4.56) and average developmental WS was 1.38 (*SD* = 0.79). Descriptive statistics for demographic data are reported in Table 1.

**Table 1:** Participant demographics

	Total, *N* = 93		AN, *n* = 38	AAN, *n* = 38	BN, *n* = 7	OSFED, *n* = 10
	M (SD)	Range	M (SD)	M (SD)	M (SD)	M (SD)
Age (years)	15.31 (1.68)	10.66–18.67	15.23 (1.88)	15.24 (1.60)	15.47 (1.74)	15.74 (1.15)
Duration of illness (months)	13.45 (12.94)	1.00–75.00	11.61 (7.58)	13.74 (14.74)	19.29 (15.26)	15.30 (19.57)
Admission BMI (kg/m^2^)	18.85 (2.31)	13.85–24.07	17.34 (1.88)	20.06 (1.82)	21.41 (1.55)	18.16 (1.84)
TWS (kgs)	5.48 (4.56)	0.00–18.15	6.55 (4.19)	13.63 (10.35)	0.25 (0.48)	2.36 (3.21)
DWS	1.38 (0.79)	0.00–4.09	1.69 (0.80)	1.01 (0.63)	0.25 (0.30)	0.78 (0.61)
EDE-Q Global Score	2.60 (1.86)	0.00–5.50	2.47 (1.79)	3.19 (1.81)	3.07 (1.61)	0.47 (0.46)
EDE-Q Restraint	2.48 (2.00)	0.00–6.00	2.55 (2.01)	3.05 (1.93)	2.31 (1.72)	0.22 (0.35)
EDE-Q Eating Concern	2.08 (1.64)	0.00–4.80	2.24 (1.55)	2.34 (1.66)	2.54 (1.50)	0.14 (0.21)
EDE-Q Weight Concern	2.74 (2.03)	0.00–6.00	2.41 (1.93)	3.49 (2.01)	3.31 (1.77)	0.80 (0.87)
EDE-Q Shape Concern	3.17 (2.20)	0.00–6.00	2.83 (2.05)	3.97 (2.09)	4.09 (1.98)	0.74 (0.99)
	***n*** **(%)**					
*Prior eating disorder treatment* ^*a*^						
Yes	26 (28.0)					
No	66 (71.0)					
*Prior medical hospitalization* ^*a*^						
Yes	36 (38.7)					
No	56 (60.2)					
*Sex*						
Female	80 (86.0)					
Male	13 (14.0)					
*Gender*						
Cisgender girl	79 (84.9)					
Cisgender boy	14 (15.1)					
Transgender boy	1 (1.1)					
*Race*						
Asian	7 (7.5)					
African American/Black	1 (1.1)					
White	72 (77.4)					
Bi/multiracial	12 (12.9)					
Declined to answer	1 (1.1)					
*Ethnicity*						
Hispanic/Latine	18 (19.4)					
Not Hispanic/Latine	75 (80.6)					

*Note. N* = 93. AN = anorexia nervosa (restricting [*n* = 33] and binge-purge [*n* = 5] subtypes combined), BMI = body mass index, BN = bulimia nervosa (full [*n* = 4] and subthreshold [*n* = 3] combined), DWS = developmental weight suppression, EDE-Q = Eating Disorder Examination Questionnaire, OSFED = other specified feeding or eating disorder, TWS = traditional weight suppression

^a^One participant missing response

Traditional WS and developmental WS were strongly correlated (*r* =.60, *p* <.001). Traditional WS did not associate with any EDE-Q outcomes. Greater developmental WS associated with lower EDE-Q Weight (*sr*^*2*^ = 0.05, *p* =.021) and Shape Concerns (*sr*^*2*^ = 0.05, *p* =.020). See Table 2 for complete parameters. Partial correlation analyses to assess the relationships between each measure of WS on EDE-Q, stratified by ED diagnosis, are reported in the Table 3.

**Table 2:** Relation of traditional and developmental weight suppression to EDE-Q admission scores

	Global Score				Restraint				Eating Concern				Weight Concern				Shape Concern			
	*F*(4,88) = 6.14^***^, *Adj. R*^*2*^ = 0.18				*F*(4,88) = 5.07^**^, *Adj. R*^*2*^ = 0.15				*F*(4,88) = 5.78^***^, *Adj. R*^*2*^ = 0.17				*F*(4,88) = 5.71^***^, *Adj. R*^*2*^ = 0.17				*F*(4,88) = 6.97^***^, *Adj. R*^*2*^ = 0.21			
	*b*	*se*	*p*	*sr* ^*2*^	*b*	*se*	*p*	*sr* ^*2*^	*b*	*se*	*p*	*sr* ^*2*^	*b*	*se*	*p*	*sr* ^*2*^	*b*	*se*	*p*	*sr* ^*2*^
TWS	− 0.03	0.02	0.074	0.03	− 0.03	0.02	0.209	0.01	−.03^t^	0.02	0.051	0.04	−.04^t^	0.02	0.060	0.03	− 0.04	0.02	0.068	0.03
Diagnosis (ref = AN)																				
AAN	0.69	0.39	0.075	0.03	0.48	0.42	0.261	0.01	0.07	0.34	0.845	0.00	1.06^*^	0.42	0.014	0.06	1.10^*^	0.45	0.016	0.05
BN	0.11	0.74	0.882	0.00	− 0.60	0.81	0.461	0.01	− 0.17	0.66	0.796	0.00	0.35	0.82	0.671	0.00	0.68	0.86	0.433	0.01
OSFED	-2.32^***^	0.62	0.000	0.12	-2.57^***^	0.69	0.000	0.13	-2.42^***^	0.55	0.000	0.17	-1.98^**^	0.69	0.005	0.07	-2.48^***^	0.73	0.001	0.10
	*F*(4,88) = 6.20^***^, *Adj. R*^*2*^ = 0.18				*F*(4,88) = 4.82^**^, *Adj. R*^*2*^ = 0.14				*F*(4,88) = 5.06^**^, *Adj. R*^*2*^ = 0.15				*F*(4,88) = 6.28^***^, *Adj. R*^*2*^ = 0.19				*F*(4,88) = 7.66^***^, *Adj. R*^*2*^ = 0.22			
	*b*	*se*	*p*	*sr* ^*2*^	*b*	*se*	*p*	*sr* ^*2*^	*b*	*se*	*p*	*sr* ^*2*^	*b*	*se*	*p*	*sr* ^*2*^	*b*	*se*	*p*	*sr* ^*2*^
DWS	− 0.44	0.25	0.074	0.03	− 0.24	0.27	0.375	0.01	− 0.28	0.22	0.213	0.01	− 0.63^***^	0.27	0.021	0.05	− 0.67^*^	0.28	0.020	0.05
Diagnosis (ref = AN)																				
AAN	0.52	0.40	0.194	0.02	0.39	0.44	0.376	0.01	− 0.03	0.36	0.936	0.00	0.81	0.44	0.067	0.03	0.84	0.46	0.073	0.03
BN	0.06	0.75	0.940	0.00	− 0.52	0.83	0.530	0.00	− 0.03	0.68	0.959	0.00	0.15	0.82	0.856	0.00	0.45	0.87	0.607	0.00
OSFED	-2.16^***^	0.60	0.001	0.11	-2.41^***^	0.67	0.000	0.12	-2.20^***^	0.54	0.000	0.15	-1.83^**^	0.66	0.007	0.07	-2.34^**^	0.69	0.001	0.10

Note. *N* = 93. AAN = atypical AN, AN = anorexia nervosa (restricting and binge-purge subtypes combined), BN = bulimia nervosa (full and subthreshold groups combined), DWS = developmental weight suppression, EDE-Q = Eating Disorder Examination Questionnaire, OSFED = other specified feeding or eating disorder, TWS = traditional weight suppression (kgs)

^***^p <.001, ^**^p <.01, ^*^p <.05, ^t^*p*<0.06

**Table 3:** Partial correlation table, WS and EDE-Q, stratified by diagnosis

	Global Score		Restraint		Eating Concern		Weight Concern		Shape Concern	
	*r*	*p*	*r*	*p*	*r*	*p*	*r*	*p*	*r*	*p*
TWS	− 0.08	0.463	0.00	0.982	− 0.07	0.407	− 0.11	0.298	− 0.10	0.334
AN, *n* = 38	− 0.12	0.486	− 0.06	0.733	− 0.11	0.521	− 0.14	0.398	− 0.16	0.351
AAN, *n* = 38	− 0.27	0.095	− 0.23	0.162	− 0.32^*^	0.049	− 0.28	0.092	− 0.23	0.161
BN, *n* = 7	− 0.034	0.941	0.099	0.833	0.13	0.786	− 0.22	0.622	− 0.09	0.850
OSFED, *n* = 10	− 0.07	0.857	0.261	0.466	0.22	0.532	0.05	0.900	− 0.30	0.396
DWS	−.20^t^	0.052	− 0.08	0.462	− 0.12	0.252	− 0.28^**^	0.009	− 0.28^**^	0.007
AN, *n* = 38	− 0.15	0.382	0.06	0.711	− 0.12	0.492	− 0.18	0.281	− 0.20	0.229
AAN, *n* = 38	− 0.22	0.190	− 0.14	0.415	− 0.14	0.387	− 0.29	0.073	− 0.26	0.120
BN, *n* = 7	− 0.56	0.190	− 0.46	0.293	− 0.51	0.238	− 0.56	0.192	− 0.53	0.219
OSFED, *n* = 10	− 0.18	0.618	0.62	.057^t^	0.417	0.231	− 0.23	0.522	− 0.44	0.204

*N* = 93. AAN = atypical AN, AN = anorexia nervosa (restricting and binge-purge subtypes combined), BN = bulimia nervosa (full and subthreshold groups combined), DWS = developmental weight suppression, EDE-Q = Eating Disorder Examination Questionnaire, OSFED = other specified feeding or eating disorder, TWS = traditional weight suppression (kgs)

^**^p <.01, ^*^p <.05, ^t^*p*<0.06

Larger dominance weights were evidenced for developmental WS compared to traditional WS across most models (Table 4); however, bootstrap sampling revealed that the difference between developmental WS and traditional WS dominance weights was not significant.

**Table 4:** General dominance weights (r^2^), DWS vs. TWS on EDE-Q admission scores

	Global Score	Restraint	Eating Concern	Weight Concern	Shape Concern
	*r* ^*2*^	*r* ^*2*^	*r* ^*2*^	*r* ^*2*^	*r* ^*2*^
TWS	0.010	0.005	0.016	0.012	0.011
DWS	0.026	0.004	0.007	0.049	0.050
Diagnosis (ref = AN)					
AAN	0.046	0.033	0.010	0.066	0.064
BN	0.004	0.004	0.004	0.004	0.009
OSFED	0.142	0.143	0.172	0.095	0.127
	95% CI	95% CI	95% CI	95% CI	95% CI
*r*^*2*^, DWS – TWS	[-0.030, 0.092]	[-0.042, 0.044]	[-0.064, 0.031]	[-0.024, 0.111]	[-0.019, 0.101]

Note. *N* = 93. AN = anorexia nervosa (restricting and binge-purge subtypes combined), BN = bulimia nervosa (full and subthreshold groups combined), DWS = developmental weight suppression, EDE-Q = Eating Disorder Examination Questionnaire, OSFED = other specified feeding or eating disorder, TWS = traditional weight suppression (kgs), WS diff. = difference in weight suppression dominance weights (DWS – TWS). Low weight anorexia nervosa (restricting subtype, binge-purge subtype) reference diagnosis. Dominance weights are *r*^*2*^, or each variable’s contribution to the unadjusted model *R*^*2*^. Confidence intervals are reported for the difference between DWS and TWS dominance weights, which were derived from 1,000 bootstrap samples from the original dataset (seed set at 123)

## Discussion

The present study assessed the incremental validity of developmental WS against traditional WS in their associations with ED symptoms in youth seeking treatment for transdiagnostic EDs. In contrast to hypotheses, developmental WS negatively associated with weight and shape concerns and was not a significantly superior correlate of ED pathology compared to traditional WS, despite accounting for greater variance in outcomes.

### Associations between WS and admission symptom severity

In contrast to previous research in adults [2] – though not surprising, given (1) our sample comprised adolescents, and (2) we calculated traditional WS irrespective of reaching adult height, both deviations from prior work – no associations emerged between traditional WS and admission ED pathology. Also in contrast to literature on traditional WS [2], which evidences a positive relation between WS and eating pathology, higher developmental WS associated with *lower* weight and shape concerns.

Generally, we expected more associations between developmental WS and eating pathology to emerge. Our lack of findings is surprising in the context of prior work, which reported smaller developmental WS ranges but still detected associations [12–15]. However, also in contrast to prior work [12–15], the current sample endorsed less pathology on the EDE-Q: nearly half (*n* = 47, 50.5%) of the current sample reported an EDE-Q global score within community norms (i.e., < 2.77 [31]). Therefore, lack of associations between developmental WS and ED pathology may be secondary to minimization and/or limited insight [32, 33]. 

The finding of an inverse relation between WS and weight and shape concerns is consistent with research on developmental WS in adults with BN [8, 12, 13]. However, this directionality contradicts positive associations observed between traditional WS and ED psychopathology across several decades of research [2]. The unique perspectives of patients lowest versus highest in developmental WS may shed light on its negative relation with body image concerns. Patients who are lower in WS are, by definition, closest to their highest past *z*-BMIs. Because fear of weight gain is a diagnostic criterion for AN and not uncommon in other EDs [18], being closer to one’s highest past weight could explain the increased weight and shape concerns found among individuals with lower WS in this sample. Alternatively, patients who are highest in developmental WS are, by definition, furthest from their highest past *z*-BMIs. For individuals who are fearful of weight gain, higher levels of developmental WS may offer psychological relief from negative body image concerns.

Dietary restraint did not associate with either WS measure at admission. This is surprising, given that high WS is often achieved via extreme caloric restriction. Possible explanations for the null finding include: (1) caregiver intervention in refeeding before initiating treatment, or (2) step-down to outpatient treatment from a higher level of care (e.g., inpatient) where caloric restriction was not possible. Given that the EDE-Q only assesses ED behaviors over the past 28 days, those highest in WS may have still engaged in extreme caloric restriction to achieve a lower weight, yet prior to the past month. Testing these hypotheses was beyond the scope of this study, given data on prior treatment was not systematically collected.

Alternatively, the EDE-Q assesses cognitive restraint, rather than true behavioral restriction [34]. Although WS requires behavioral restriction, it does not necessitate cognitive restriction, which patients with EDs are likely to minimize and/or underreport [35–39]. 

### Comparative utility of traditional WS versus developmental WS

Despite associating with more outcomes and possessing larger dominance weights compared to traditional WS, developmental WS was not a significantly superior correlate of ED pathology. This finding not only contradicts the premise upon which developmental WS was initially proposed [8] but also contrasts with findings in recent work [12–15]. Notably, the means for developmental and traditional WS were larger and smaller, respectively, compared to those reported in prior work [12–15]. This difference would, hypothetically, magnify differences between the two measures. The opposite finding may instead be due to a restricted range of our dependent variable, which may have limited our ability to detect differential associations. Reasons for a restricted range of EDE-Q score may include, as noted above, minimization.

Regardless, results preliminarily suggest that a *z*-BMI approach to calculating WS is more sensitive to body image concerns among adolescents with EDs; however, when minimization of concerns is present, neither WS calculation is an adequate proxy for other cognitive symptoms (e.g., restraint). Given that this is the first study to explore associations between different calculations of WS in youth with AN, replication is needed. However, because developmental WS is sensitive to developmental considerations, this calculation is still recommended for youth when growth history is available.

### Strengths, limitations, and future directions

The current study is the only study to date to examine the incremental validity of developmental WS versus traditional WS in adolescents with transdiagnostic EDs. Another notable strength is our use of growth charts to retrieve weight history data. Prior literature has used self-reported highest premorbid weight, height, and age to calculate developmental WS [12–15]. However, historical self-report can be flawed, and highest premorbid *z*-BMI does not necessarily coincide with highest past weight. Our approach of including BMIs that correspond to an individual’s highest premorbid percentile addresses both issues, thus representing stronger methodology compared to prior work.

We highlight that the goal of our study is to provide data to support the incremental validity of developmental WS in youth. Its focus on analytic methodology is a strength; however, as a result, its lack of focus on clinical relevance may be perceived as a weakness. We argue that establishing the validity and reliability of new methods, such as those used to calculate developmental WS, is a necessary first step before applying novel calculations to answer rigorous clinical questions.

Our study is limited by the nature of data available; namely, cross-sectional EDE-Q scores. Cross-sectional data precludes our ability to test the predictive value of developmental WS in youth. Moreover, the EDE-Q has been increasingly recognized as flawed for individuals across the gender [40] and diagnostic spectrum [17]. Therefore, an important step in future work will be validating developmental WS against alternative measures of eating pathology (e.g., The Eating Pathology Symptoms Inventory [41, 42]) measured longitudinally.

Because we were interested in validating developmental WS in youth, we did not aim to explore its interactions with other clinical variables (e.g., diagnosis); however, we encourage such steps in future work. Additionally, values for developmental WS in the current sample followed a normal distribution, thereby supporting the use of *z*-scores in calculating WS. However, in cases where the distribution is skewed or outliers are present, the use of percent median BMI to calculate WS may be more apt, as BMI *z*-scores may attenuate differences at the extremes of the distribution. We also recoded negative traditional and developmental WS values to 0, based on the formula for calculating WS set forth in the literature [1, 8]. According to this calculation, an individual with whose current weight is 10 kg higher than highest premorbid weight would have the same WS as someone whose current weight equals their highest premorbid weight (i.e., 0). Although this represents a shortcoming of the original calculations proposed [1, 8] rather than that of our paper, future work may investigate whether there is merit in *not* recoding negative WS values. Finally, although set-point theory – or the notion that each individual has a genetically predetermined weight range that their body strives to maintain [43] – was influential in the conceptualization of WS for adults [1], it is unclear whether the same biobehavioral bind applies to children and adolescents whose historical BMIs fall in the “overweight” or “obese” ranges. Preliminary evidence suggests that those with atypical AN may recover with 25% less weight gain [44]; however, more research in this area is needed. Although this line of inquiry is beyond the scope of the current study, developmental WS offers a novel approach to probe this hypothesis in future work.

## Conclusions

To conclude, in this sample of youth with transdiagnostic EDs, developmental WS correlated with pretreatment body image concerns. Though replication with larger samples and longitudinal data is needed, our results preliminarily support the use of developmental WS over traditional WS in youth.

## Data Availability

The data that support the findings of this study are available from the corresponding author upon reasonable request.

## Abbreviations

AN: Anorexia nervosa
BMI: Body mass index
BN: Bulimia nervosa
DSM-5: Diagnostic and Statistical Manual of Mental Disorders, 5th ed
ED: Eating disorder
EDE-Q: Eating Disorder Examination Questionnaire
WS: Weight suppression
